# Anti-Irritant and Anti-Inflammatory Effects of DHA Encapsulated in Resveratrol-Based Solid Lipid Nanoparticles in Human Keratinocytes

**DOI:** 10.3390/nu11061400

**Published:** 2019-06-21

**Authors:** Simona Serini, Roberta Cassano, Enrica Facchinetti, Gaia Amendola, Sonia Trombino, Gabriella Calviello

**Affiliations:** 1 Institute of General Pathology, Università Cattolica del Sacro Cuore, Largo F. Vito, 00168 Roma, Italy; simona.serini@unicatt.it (S.S.); dridinetti@gmail.com (E.F.); gaia_amendola@libero.it (G.A.); 2 Fondazione Policlinico Universitario A. Gemelli IRCCS, Largo F. Vito, 00168 Roma, Italy; 3 Department of Pharmacy, Health and Nutritional Sciences, Università della Calabria, Arcavacata di Rende, 87036 Cosenza, Italy; roberta.cassano@unical.it

**Keywords:** docosahexaenoic acid, human keratinocytes, inflammasome, inflammation, irritation, sodium dodecyl sulfate, solid lipid nanoparticles

## Abstract

We recently found that the dietary long chain omega-3 polyunsaturated fatty acid (LC-ω-3 PUFA), docosahexaenoic acid (DHA), showed enhanced antineoplastic activity against colon cancer cells if encapsulated in resveratrol-based solid lipid nanoparticles (RV-SLNs). In the present study, we investigated whether the DHA enclosed in RV-SLNs (DHA-RV-SLNs) could have the potential of attenuating irritation and inflammation caused by environmental factors at the skin level. To this aim, we used two keratinocyte lines (HaCaT and NCTC 2544 cells) and exposed them to the cytotoxic action of the surfactant, sodium dodecyl sulfate (SDS), as an in vitro model of irritation, or to the pro-inflammatory activity of the cytokine TNF-α. We found that DHA enclosed in RV-SLNs significantly enhanced its ability to contrast the cytotoxic effect of SDS and to inhibit the SDS- and TNF-α-induced production of the inflammatory cytokines IL-1β, IL-6, and 1 MCP-1, in the two keratinocyte cell lines, as well as the NLRP3 inflammasome activation. Moreover, it more efficiently reduced the upsurge of reactive oxygen species (ROS) levels obtained in the presence of a pro-oxidant (H_2_O_2_). Overall, our findings suggest the possibility that a sustained dietary supplementation with DHA-RV-SLNs could efficiently protect skin from the pro-irritant and pro-inflammatory activity of environmental attacks.

## 1. Introduction

Long chain omega-3 polyunsaturated fatty acids (LC-ω-3 PUFAs) of marine origin are components of our diet showing multiple healthy effects, including powerful anti-inflammatory activities [1], as demonstrated in several pathological settings, including cardiovascular [2], neoplastic [3], and neurodegenerative [4] diseases. We previously found [5] that the two main polyunsaturated ω-3 fatty acids, eicosapentaenoic acid (EPA, 20:4 ω-3) and docosahexaenoic acid (DHA, 22:6 ω-3), were able to modulate the inflammatory phenotype of mononuclear cells isolated from peripheral blood of Alzheimer’s disease patients, reducing the levels of Interleukin (IL)-1β and IL-6 and increasing those of the anti-inflammatory cytokine, IL-10.

The anti-inflammatory activity of LC-ω-3 PUFA has been in part related to their capacity to displace the long-chain ω-6 PUFA arachidonic acid (AA) from cellular membranes, thus inhibiting the formation of AA metabolic oxidative products with high pro-inflammatory potential [6]. On the other hand, the LC-ω-3 PUFA ability to inhibit the inflammatory response has been more directly associated to some of their metabolic derivatives (such as resolvins, maresins, or protectins) showing powerful anti-inflammatory and pro-resolving activities [7]. Acute inflammation represents the physiological reactive response against infections and tissue damage, and its main role is eliminating the offending agents and inducing regeneration of damaged tissue. However, an inappropriate, excessive, or long-lasting inflammatory process may lead to detrimental effects and to the development of a series of inflammation-related human diseases, including autoimmune diseases [8], allergic reactions [9], and cancer [10].

One of the body districts more easily subject to acute and chronic inflammation is skin, which represents the barrier protecting our body from harmful environmental factors. Inflammatory processes represent the main component of a series of skin disorders [11,12], and the possibility of preventing/attenuating skin inflammation with a treatment with LC-ω-3 PUFAs has been explored in psoriasis [13], atopic dermatitis, and photoaging [14]. To this aim, the topical application of these fatty acids has often been used, since it allows high concentrations of these fatty acids in the target tissue to be reached. However, a constant dietary supplementation with LC-ω-3 PUFAs would allow an increase of the levels of these fatty acids in all the cell membranes inside the body, including the cells of innate and adaptive immunity, deeply involved in the development of some skin disorders, and could ensure the sustained tissue availability of these fatty acids independently from their frequent topical applications. In recent years, to make the delivery to tissues of dietary supplemented LC-ω-PUFAs more efficient, much effort has been put in to trying to increase the bioavailability of these dietary fatty acids and to ensure high levels of them in cell membranes. In fact, EPA and DHA, which are more bioactive among the LC-ω-3 PUFAs, are also highly peroxidable due to the presence of multiple double bonds in their carbon chains. In the last few years, among the possibilities explored to prevent their oxidative degradation, both before their intake (to prolong their shelf-life) and during the absorbance and delivery to the tissues of interest, their inclusion in micro- and nano-particles has gained increasing attention [15]. Recently, to enhance their incorporation in cells, and prevent their peroxidation and degradation, we constructed solid lipid nanoparticles (SLNs) [16], which are particles with a spherical shape, a nanometer size range, and are dispersed in water or in aqueous surfactants [17,18]. These nanoparticles were shown to be very useful for the incorporation of chemically labile active ingredients by protecting them against degradation. In particular, in our previous work, we obtained SLNs with a lipid matrix based on resveratrol (RV) esterified to stearic acid, and encapsulating either linolenic acid (LNA, 18:3 ω-3) or DHA [16]. We found that when we administered LNA or DHA encapsulated in the RV-based SLNs to colon cancer cells in vitro, the anti-proliferative effect of the fatty acids was significantly enhanced as compared to their non-encapsulated forms.

In the present work, we sought to investigate whether the encapsulation in these RV-based SLNs could also improve the anti-inflammatory activity of DHA in normal skin cells. We chose to use the DHA-containing SLNs, since we had previously found that this fatty acid exerted powerful anticarcinogenic activity in UV-treated normal human keratinocytes [19], as well as in metastatic and non-metastatic melanoma cells [20,21,22]. This was interesting, since the development of both non-melanoma and melanoma skin cancers has been associated to chronic inflammatory processes of the skin [23]. Irritation is a predominant feature of skin inflammatory disorders, and may be caused by physical, mechanical, or chemical agents [24]. Therefore, we analyzed the anti-irritant and anti-inflammatory effect of DHA and DHA-containing RV-based SLNs (DHA-RV-SLNs) in human immortalized HaCaT and NCTC 2544 keratinocyte lines known to show a different degree of differentiation [19,25]. They represent a suitable in vitro model of the basal and suprabasal layers of the epidermis. In these cells, we analyzed the production of IL-1β and IL-6 and of the cytokine, monocyte chemoattractant protein-1 (MCP-1), induced by SDS and by the pro-inflammatory cytokine Tumor Necrosis Factor (TNF)-α. In these conditions, we also evaluated the cellular reactive oxygen species (ROS) formation and the activation of NLR Family Pyrin Domain Containing 3 (NLRP3) inflammasome, which has been in part related to an increased cellular ROS production [26].

## 2. Materials and Methods

### 2.1. Reagents and Solvents

Chloroform, methanol, tetrahydrofuran, and butanol were purchased from Carlo Erba Reagenti (Milan, Italy). Chloroform was purified through standard procedures. Resveratrol, stearic acid (MM = 284.48 g/mol), *N*,*N*′-Dicyclohexylcarbodiimide (DCC), 4-Dimethylaminopyridine (DMAP), Tween 80, sodium taurocholate hydrate, CDCl_3_, trichloroacetic acid (TCA), thiobarbituric acid (TBA), and butylated hydroxytoluene (BHT) were purchased from Sigma-Aldrich (Sigma Chemical CO, St. Louis, MO, USA).

### 2.2. RV-SLNs Preparation

The resveratrol-stearate solid lipid nanoparticles (RV-SLNs), either empty or loaded with DHA, were prepared successfully through the microemulsion technique as previously described [16,27,28,29,30,31]. In a previous work of ours [16], we evaluated the average diameter, polydispersity index (PI), dimensions, shape of the RV-SLNs, and analyzed the percentage of DHA encapsulation (EE) using a UV-vis spectrophotometer, obtaining a value of 100%, thus demonstrating a high chemical affinity of DHA for the ester composing the nanoparticles.

### 2.3. Cell Lines

The human immortalized keratinocytes, HaCaT, were obtained by the American Type Culture Collection (ATCC, Rockville, MD, USA). The human immortalized keratinocytes, NCTC 2544, were a kind gift from Dr. R. De Bellis (Università di Urbino, Italy). Both the cell lines were maintained in Dulbecco’s modified minimal essential medium (DMEM) containing 2 mM glutamine and antibiotics (100 U/mL penicillin and 100 μg/mL streptomycin) in the presence of 10% fetal bovine serum. Cells were maintained in the exponential growth phase by trypsinization and seeding at the concentration of 3 × 10^5^ cells/mL two times/week.

### 2.4. Reagents and Treatments

Docosahexaenoic acid (DHA, 22-6 ω-3) was purchased from Sigma-Aldrich (Sigma, St. Louis, MO, USA). The free fatty acid was added to the culture medium at the concentration of 10 and 30 µM from an absolute ethanol stock solution (10 mM). In this case, control cells were treated with the same amounts of vehicle alone (never exceeding the concentration of 0.5%, v/v in the culture medium). For the experiments with the solid lipid nanoparticles (SLNs), stock solutions of both the empty RV-SLNs and the DHA-containing RV-SLNs were prepared using cell culture medium. Since we had previously determined the incorporation efficiency of the DHA-RV-SLNs [16], we treated the cells with DHA-RV-SLN stock solution volumes in order to obtain the same final concentration of the free DHA (10 and 30 µM). In this case, control cells were treated with the same volumes of culture medium containing the empty RV-SLN.

We chose SDS concentrations ranging from 5 to 50 µg/mL, since in preliminary experiments, we found that higher concentrations (75–100 µg/mL) induced reduced cell viability to values lower than 5% (data not shown). We stimulated keratinocytes with 50 ng/mL TNF-α since from preliminary experiments, we found that this concentration was able to induce the maximal effect on the secretion of IL-1β, IL-6, and MCP-1 without inducing cell cytotoxicity (data not shown).

### 2.5. Analysis of Cell Viability

The HaCaT and NCTC 2544 keratinocytes were seeded at the concentration of 3 × 10^4^ cells/well in a 24-well multiwell plate (three wells for each concentration studied). After 24 h cell culture, medium was removed and replaced with fresh culture medium containing or not sodium dodecyl sulfate (SDS) at increasing concentrations (0–50 µg/mL), or pure DHA (30 µM) or DHA-SLN (30 µM), alone and in combination. After 48 h of treatment, cells were washed in phosphate buffer (PBS, pH 7.4), trypsinized, centrifuged, and counted by using a Neubauer chamber. Cell viability was evaluated through the Trypan blue exclusion method [32].

### 2.6. Analysis of Inflammatory Cytokine Production

HaCaT and NCTC 2544 cells were seeded at the concentration of 5 × 10^3^ cells/mL in a 96-well multiwell culture plate. When cells reached the 80% confluence, culture medium was removed and replaced with fresh culture medium containing or not SDS (50 µg/mL), TNF-α (50 ng/mL), pure DHA (10 and 30 µM), or DHA carried by SLNs at the same concentrations of pure DHA, alone or in combination. At the indicated time points (48 and 72 h), cell supernatants were collected, centrifuged to remove cell debris, and stored at −80 °C until analysis. The production of cytokines was performed by using commercially available Enzyme-linked immunosorbent assay (ELISA) kits (Biolegend, San Diego, CA, USA) following the manufacturer’s instructions. The minimum detectable amounts of IL-1β, IL-6, and MCP-1 were 0.5, 4, and 3.9 pg/mL, respectively.

### 2.7. Analysis of ROS Production

HaCaT and NCTC 2544 cells were seeded at the concentration of 1 x 10^5^ cells/well in a 24-well multiwell culture plate. After 24 h, cell culture medium was removed and replaced with fresh culture medium containing or not SDS (50 µg/mL), TNF-α (50 ng/mL), pure DHA (10 and 30 µM), or DHA delivered through SLNs at the same concentration, alone and in combination. After 24 h, cells were washed with PBS and then incubated in the presence of the fluorogenic substrate, 6-carboxy-2′,7′-dihydrodichlorofluorescein diacetate (DCF, 50 µM), at 37 °C in the dark for 30 min. In this form, the substrate is not fluorescent and, following the cell uptake, it is de-esterified in the presence of ROS and transformed into the fluorescent compound, 2′,7′-dichlorofluorescein. The fluorescence emitted was measured by a plate cytofluorimeter (Cytofluor 2300/2350 Fluorescence Measurement System, Millipore Corp., Bedford, MA) with an excitation wavelength of 485 nm and an emission wavelength of 530 nm. In order to analyze the effect of the compounds in the presence of a pro-oxidant stimulus, cells were then exposed to 100 µM H_2_O_2_ for a further 15 min at 37 °C in the dark. This concentration has been used since in preliminary experiments (data not shown), H_2_O_2_ was found to induce a maximal pro-oxidant effect without inducing cell death.

### 2.8. Analysis of NLRP3 Inflammasome Activation by Western Blotting

HaCaT cells were seeded in 60 mm Petri dishes at the concentration of 3 × 10^5^ cells/ mL. After 24 h, cell culture medium was removed and replaced with fresh culture medium containing or not TNF-α (50 ng/mL), pure DHA (30 µM), or DHA-containing SLNs at the same concentrations, alone and in combination. After 48 h of treatment, cells were washed in PBS, trypsinized, and centrifuged at 225× *g* for 5 min to obtain the cell pellet. Total cell lysates were prepared according to Serini et al. [20]. Briefly, 100 µL of cold lysis buffer [1 mM MgCl_2_, 350 mM NaCl, 20 mM 4-(2-hydroxyethyl)-1-piperazineethanesulfonic acid (HEPES), 0.5 mM ethylenediaminetetra-acetic acid (EDTA), 0.1 mM ethylene glycol tetraacetic acid (EGTA), 1 mM Na_4_P_2_O_4_, 1 mM phenylmethylsulfonylfluoride, 1 mM aprotinin, 1.5 mM leupeptin, 20% glycerol, 1% 4-Nonylphenyl-polyethylene glycol (NP-40)] were added to each sample. The samples were then incubated for 30 min on ice and then centrifuged (13,000× *g* for 15 min at 4 °C) to remove cell debris. The protein concentration of the lysates was determined through the Bradford method using the Biorad assay (Hercules, CA, USA). In total, 80 µg of protein were separated on a SDS-polyacrylamide gel and then transferred to a PVDF membrane. The membrane was blocked for 1 h at room temperature with 5% milk powder in TBST (PBS containing 0.05% Tween 20) and then incubated overnight at 4 °C with the specific antibodies against NLRP3 (MAB7578, clone #768319, R&D Systems, Minneapolis, MN, USA), ASC (clone N-15-R: catalog # sc-22514-R, Santa Cruz Biotechnology, Santa Cruz, CA, USA), and cleaved caspase-1 (clone C-20: sc-515, Santa Cruz Biotechnology). As a loading control, membranes were incubated in the presence of stripping solution (for 10 mL solution: In total, 10 mL of 10% SDS, 312 µL HCl, and 6.5 µL β-mercaptoethanol) at 50 °C for 30 min, washed in TBST, blocked, and incubated with a β-actin antibody (clone AC40, catalog # A-4700, Sigma-Aldrich) at a 1:1000 dilution. After the incubation with a secondary anti-rabbit (for ASC and cleaved caspase-1), anti-rat (NLRP3), or anti-mouse (for β-actin), immunocomplexes were visualized by using the chemiluminescence detection system (GE Healthcare Life Sciences, Pittsburgh, PA, USA) and quantitated through densitometric analysis.

### 2.9. Statistical Analysis

Data were analyzed by the one-way analysis of variance (one-way ANOVA), followed by Tukey’s test. Differences were considered significant at *p* < 0.05.

## 3. Results and Discussion

Surfactants can penetrate the skin and interact with proteins and lipids, inducing epidermal barrier damage, skin dryness, erythema, irritation, itching, and thickening [33,34,35,36,37]. These compounds are components of cleansing formulations due to their ability to solubilize hydrophobic compounds (oils, sebum, dirt) present on the skin, thus allowing effective washing away of these substances [33,38]. In particular, to assess the irritant potential of surfactants, evaluation of cytotoxicity in vitro and the production of inflammatory cytokines has been widely used [39]. The surfactant, SDS, is one of the most used reference substances in experiments evaluating pro- or anti-irritatives, as well as pro-and anti-inflammatory effects of other compounds that are potentially protective or irritative/pro-inflammatory themselves [40,41,42,43,44]. In order to evaluate the protective effect of DHA (free or encapsulated in nanoparticles) against skin irritation/inflammation, we took advantage of the reported cytotoxic effect of SDS towards normal cultured keratinocytes [39], and of the well-known ability of the cytokine, TNF-α, in inducing a pro-inflammatory status, as in vitro models of skin irritation and inflammation.

### 3.1. Effect of DHA and DHA-RV-SLNs on the SDS Growth Inhibiting Effect in Cultured Keratinocytes

In order to verify if, and at what concentrations, SDS was able to induce cytotoxicity in HaCaT and NCTC 2544 keratinocytes in our experimental conditions, we preliminarily treated the cells with increasing concentrations of SDS (5–50 µg/mL) and evaluated their ability to inhibit cell growth after 48 h of treatment (Supplementary Materials Figure S1). We found that 5 and 10 µg/mL SDS did not significantly modify keratinocyte growth, whereas 50 µg/mL SDS markedly reduced it, with HaCaT growth that was inhibited by 93.7% (vs. control, *p* < 0.05) and NCTC 2544 by 43.5% (vs. control, *p* < 0.05) (Supplementary Materials Figure S1A,B).

This finding confirmed the results previously reported by Lawrence et al. [45] in keratinocytes from rat sublingual mucosa at concentrations (82.5 µg/mL) similar to those exerting a cytotoxic effect in our experimental model. Powerful cytotoxic effects were also reported more recently by other authors that exposed the same keratinocytes used by us (HaCaT and NCTC 2544) to concentrations of SDS higher than that used by us, but for shorter periods [43,46]. In particular, Zhang et al. [46] exposed HaCaT cells to 100 µM SDS for 24 h, and Parodi et al. [43] exposed NCTC 2544 cells to 0.5 mM-18 mM SDS for just 3 h.

**Figure 1:**
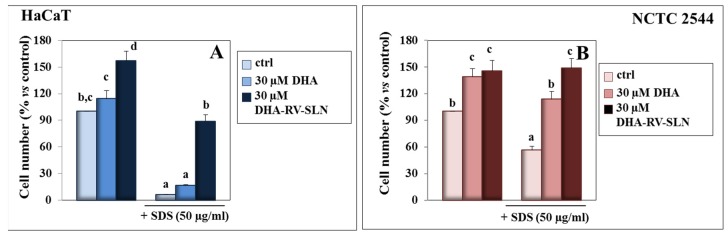
Effect of free DHA and DHA-RV-SLNs on SDS-induced cytotoxicity in HaCaT and NCTC 2544 keratinocytes. HaCaT (**A**) and NCTC 2544 (**B**) cells were treated with 30 µM free DHA or 30 µM DHA-RV-SLNs in the absence or in the presence of 50 µg/mL SDS for 48 h. Data are the means ± SD of three different experiments. Values not sharing the same superscript letter are significantly different (*p* < 0.05, one-way ANOVA followed by Tukey’s test).

For the following experiments, we decided to use DHA at concentrations ≤30µM, since they did not exert any cytotoxic effect on the two keratinocyte cell lines, differently from the higher concentrations (50–100 µM), which induced clear cytotoxic effects when tested with the Trypan Blue exclusion test (data not shown). On the contrary, we observed (Figure 1) that the treatment with free DHA at 30 µM enhanced the growth of both the HaCaT and NCTC 2544 keratinocytes, even though this effect was significant only in the NCTC 2544 cells (cell number increase: 39.2% vs. control, *p* < 0.05). We observed a more pronounced protective effect when 30 µM DHA was carried by RV-SLNs in both the cell lines (Figure 1A,B). When the HaCaT keratinocytes were simultaneously exposed to SDS and DHA, either in the free fatty acid or encapsulated form, a significant inhibition of the SDS-induced cytotoxic effect was observed only when DHA was administered as DHA-RV-SLNs. On the contrary, we also observed that free DHA was able to revert the inhibitory effect of SDS on cell growth in NCTC 2544 keratinocytes. These findings are interesting, since, for the first time, they showed a protective activity of DHA against the cytotoxic action of surfactants in human cultured keratinocytes, and also since they demonstrated that DHA had a more pronounced effect when it was encapsulated in RV-SLNs. (Figure 1A,B). The higher protective effect that DHA-RV-SLNs showed in HaCaT cells as compared to NCTC 2544 cells (about 14.1 fold and about 2.6 fold increase in cell number, respectively, vs. SDS-treated cells) is particularly interesting. In fact, HaCaT cells, due to their higher intrinsic differentiation degree, represent a cellular model for the superficial suprabasal layers of the epidermis, which are particularly exposed to environmental hazards, including the irritants contained in detergents. On the other hand, NCTC 2544 cells have been considered a suitable model for the more undifferentiated basal layer of the epidermis [19], which are less exposed to environmental agents. Since an increased dietary intake of LC-ω-3 PUFAs induces their enhanced accumulation in all the cell membranes throughout the body, including the skin district [47,48], our findings suggest that high levels of DHA could be obtained in epidermal keratinocytes in vivo by a dietary supplementation with the DHA-RV-SLNs. This, in turn, could represent an effective protection against environmental hazards. This hypothesis is supported by our recent findings [16] obtained in colon cancer cells cultured in vitro, where we demonstrated that DHA enclosed in the RV-SLNs resulted in more efficient incorporation than free DHA in the cells. It should be underlined that, since DHA is a highly peroxidable compound, the cytoprotective effect observed may also be related to the presence of the antioxidant, RV, in the lipid matrix of the SLN used to encapsulate DHA. In fact, we showed previously [16] that these RV-SLNs had the ability to act as efficient antioxidants, due to their lipidic matrix component RV. This suggests that, once encapsulated, DHA may be better preserved from its oxidative degradation and, thus increase its beneficial effects.

### 3.2. Effect of DHA and DHA-RV-SLNs on the Basal and SDS-Induced IL-1β Production in Cultured Keratinocytes

**Figure 2:**
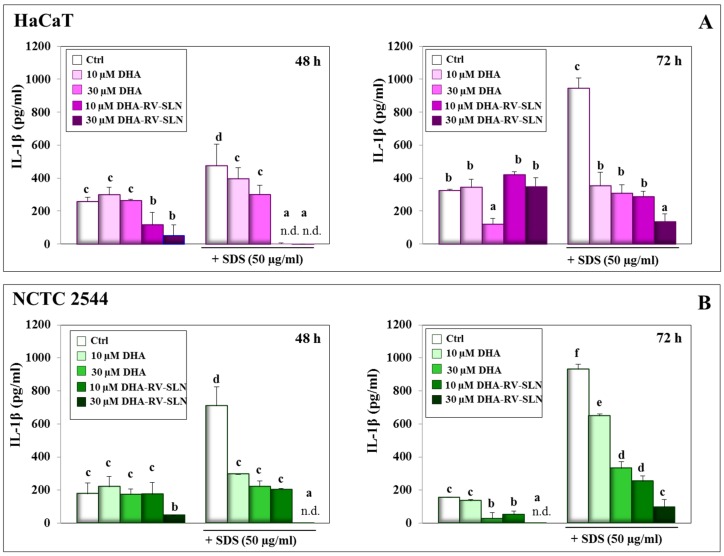
IL-1β production in HaCaT and NCTC 2544 keratinocytes treated with SDS alone and in combination with DHA delivered or not through RV-SLNs. (**A**): HaCaT cells; (**B**): NCTC 2544 cells. The cells were treated with free DHA or DHA-RV-SLNs (10 or 30 µM) in the absence or in the presence of 50 µg/mL SDS for 48 (left panels) or 72 h (right panels). Data are the means ± SD of three different experiments. Values not sharing the same superscript letter are significantly different (*p* < 0.05, one-way ANOVA followed by Tukey’s test).

Keratinocyte damage induced by surfactants, such as SDS, in the skin has been reported to induce the release of inflammatory cytokines [49]. On this basis, we evaluated the basal and SDS-induced production of some pro-inflammatory cytokines by HaCaT and NCTC 2544 cells, with the main aim to investigate whether this production could be altered by DHA (either in its free form or enclosed in RV-SLNs). In fact, the anti-inflammatory activity of omega-3 fatty acids has been largely recognized also at the skin level [50]. We firstly evaluated the IL-1β production since it is known as one of the major epidermal pro-inflammatory cytokines, and its overexpression has been associated to the progression of several skin inflammatory conditions and cancer [51]. We observed that the treatment of both the keratinocytes with free DHA for 48 h was not sufficient to reduce the IL-1β basal cell production, which, however, was significantly decreased after 72 h at 30 μM DHA (HaCaT cells: 62.8% inhibition vs. control, *p* < 0.05; NCTC 2544 cells: 82.8 % inhibition vs. control *p* < 0.05) (Figure 2A,B). The higher anti-inflammatory efficacy of DHA observed in the basal layer-like NCTC 2544 cells is noteworthy, since it is known that less differentiated basal cells from the epidermis are more susceptible than the more differentiated superficial keratinocytes to the carcinogenic effect of a pro-inflammatory environment. When DHA was carried by RV-SLNs, it inhibited more precociously (after 48 h of treatment) the IL-1β basal production in both the cells. In HaCaT cells, however, the effect was transient (10 µM DHA-SLN, 55.3% inhibition; 30 µM DHA-SLN, 80.1% inhibition, *p* < 0.05) and was not observed any more at 72 h. On the contrary, in NCTC2544 cells, only 30 μM DHA-RV-SLNs significantly decreased the IL-1β basal cell production after 48 h (74.2% inhibition, *p* < 0.05), and at 72 h, the IL-1β production was completely abolished. Again, our results suggest that when DHA is delivered through RV-SLNs, it may elicit a more rapid protective response, ascribable probably to a more rapid and/or more efficient DHA absorption by keratinocytes.

The treatment with SDS (50 µg/mL) induced a significant and time-dependent increase in IL-1β production (48 h: 84% increase; 72 h: 190.7% increase vs. control, *p* < 0.05) in HaCaT cells (Figure 2A). Similarly, in NCTC 2544 cells (Figure 2B), SDS induced a significant and time-dependent increase in the cytokine production (48 h: 302.9% increase; 72 h: 501.7% increase vs. control, *p* < 0.05). These results are in agreement with those obtained previously in different in vitro [52] and in vivo [53] experimental models. In particular, Pauloin et al. [52] utilized a model of epithelial corneal cells in vitro and observed an increased production of IL-1β and IL-8 following the treatment with SDS at concentrations (30–70 µg/mL) very similar to those used by us in the present study. Szel et al. [53] used SKH-1 mice exposed to SDS-induced acute skin irritation, and reported an increased production of different pro-inflammatory cytokines, including IL-1α, IL-1β, and TNF-α.

The concomitant treatment with SDS and DHA, either as free fatty acid or carried by RV-SLNs, significantly reduced the effect of SDS on IL-1β production. Particularly, DHA enclosed in RV-SLNs was much more efficacious than free DHA (HaCaT cells, 30 µM DHA: 36.8% inhibition; NCTC 2544 cells, 30 µM DHA: 68.8% inhibition, at 48 h, *p* < 0.05), being able to completely inhibit the IL-1β production at 48 h in both cells (Figure 2A,B). This higher efficacy was also observed at 72 h.

### 3.3. Effect of DHA and DHA-RV-SLNs on the Basal and SDS-Induced IL-6 Production in Cultured Keratinocytes

**Figure 3:**
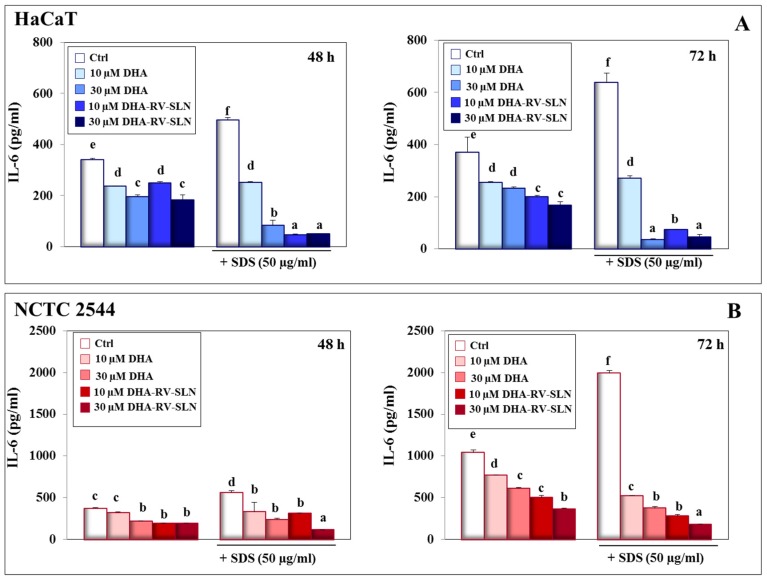
IL-6 production in HaCaT and NCTC 2544 keratinocytes treated with SDS alone and in combination with DHA delivered or not through RV-SLNs. (**A**): HaCaT cells; (**B**): NCTC 2544 cells. The cells were treated with free DHA or DHA-RV-SLNs (10 or 30 µM) in the absence or in the presence of 50 µg/mL SDS for 48 (left panels) or 72 h (right panels). Data are the means ± SD of three different experiments. Values not sharing the same superscript letter are significantly different (*p* < 0.05, one-way ANOVA followed by Tukey’s test).

IL-6 is another of the main pro-inflammatory cytokines released by activated keratinocytes during the pathogenesis of inflammation-related disorders, including psoriasis [54], and other skin disorders, such as atopic dermatitis [55], neutrophilic dermatosis [56], and skin photo-toxicity [57]. The basal IL-6 production was significantly inhibited by DHA and DHA-RV-SLNs in both the keratinocytes at 48 h (Figure 3A). SDS-induced a considerable increase in the production of this cytokine (HaCaT cells, 48 h: 46.2% increase, 72 h: 72.2% increase vs. control, *p* < 0.05; NCTC 2544 cells, 48 h: 51.2% increase, 72 h: 91.0% increase vs. control, *p* < 0.05) (Figure 3A,B). Moreover, free DHA was very efficacious in inhibiting SDS-induced IL-6 production in a dose-dependent manner at both time points and in both the cells. Once again, the effect of DHA-RV-SLNs was significantly higher than that exerted by free DHA, particularly at the lowest concentration used (10 µM) (Figure 3A,B). This result is particularly interesting since the delivery of DHA through this nanoformulation is potentially able to induce a powerful anti-inflammatory effect even at low concentrations.

### 3.4. Effect of DHA and DHA-RV-SLNs on the Basal and SDS-Induced MCP-1 Production in Cultured Keratinocytes

**Figure 4:**
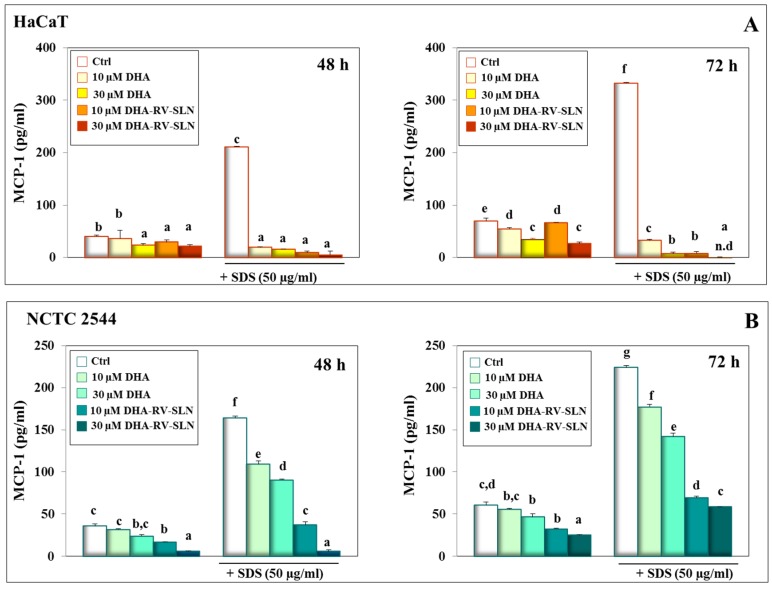
MCP-1 production in HaCaT and NCTC 2544 keratinocytes treated with SDS alone and in combination with DHA delivered or not through RV-SLNs. (**A**): HaCaT cells; (**B**): NCTC 2544 cells. The cells were treated with free DHA or DHA-RV-SLNs (10 or 30 µM) in the absence or in the presence of 50 µg/mL SDS for 48 (left panels) or 72 h (right panels). Data are the means ± SD of three different experiments. Values not sharing the same superscript letter are significantly different (*p* < 0.05, one-way ANOVA followed by Tukey’s test).

The cytokine, MCP-1, is known for its ability to recruit monocytes at the skin level, and it is considered to play a very important role in inducing a chronic inflammatory status in this tissue [58]. Both free DHA and DHA-RV-SLNs exerted a powerful inhibiting effect on MCP-1 production, both in basal conditions and in the presence of SDS (Figure 4A). In particular, DHA-RV-SLNs at the higher concentration used (30 µM) reduced the amount of SDS-induced MCP-1 production at undetectable levels in HaCaT cells after 72 h of treatment (Figure 4A, right panel). A significant and dose-dependent reduction in SDS-induced MCP-1 levels was also observed in NCTC 2544 cells, even if it was smaller (72 h and 30 µM DHA-RV-SLNs: 73.9% inhibition vs. control, *p* < 0.05) (Figure 4B, right panel). The greater MCP-1 reducing effect of both free DHA and DHA-RV-SLNs in the suprabasal layer-like HaCaT cells is noteworthy, since the more superficial keratinocytes are the most exposed to environmental pro-inflammatory agents. Moreover, the MCP-1 reducing effect of DHA-RV-SLNs was always more powerful than that observed in the presence of free DHA at both the concentrations used, and in both the cells (Figure 4A,B).

### 3.5. Effect of Free DHA and DHA-RV-SLNs on ROS Production Induced by SDS and TNF-α in Human Keratinocytes In Vitro

**Figure 5:**
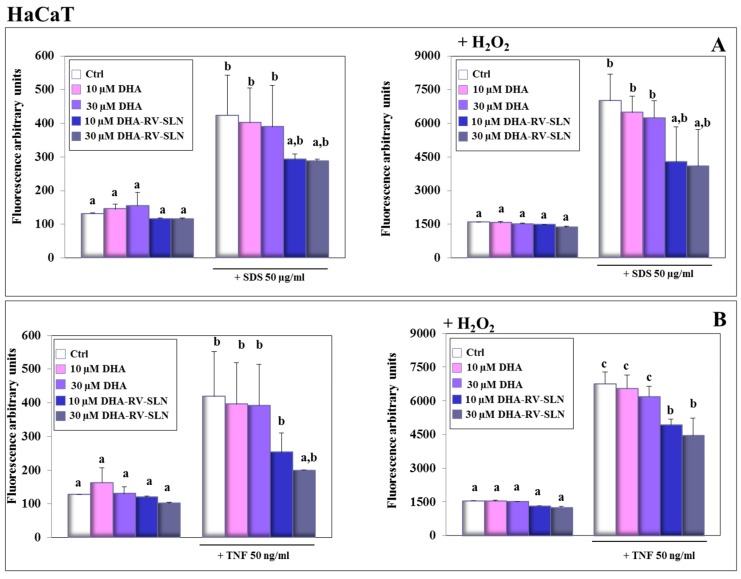
Effect of free DHA and DHA-RV-SLNs on ROS production in HaCaT keratinocytes treated with SDS or TNF-α. The cells were treated with free DHA or DHA-RV-SLNs (10 or 30 µM) in the absence or in the presence of 50 µg/mL SDS (**A**) or 50 ng/mL TNF-α (**B**) for 24 h, in basal conditions (left panels) or in the presence of 100 µM H_2_O_2_ as a pro-oxidant stimulus (right panels). Data are the means ± SD of three different experiments. Values not sharing the same superscript letter are significantly different (*p* < 0.05, one-way ANOVA followed by Tukey’s test).

**Figure 6:**
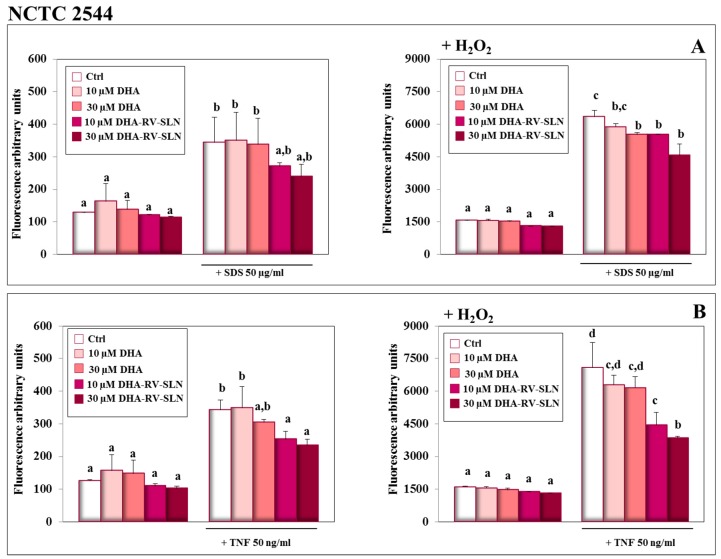
Effect of free DHA and DHA-RV-SLNs on ROS production in NCTC 2544 keratinocytes treated with SDS or TNF-α. The cells were treated with free DHA or DHA-RV-SLNs (10 or 30 µM) in the absence or in the presence of 50 µg/mL SDS (**A**) or 50 ng/mL TNF-α (**B**) for 24 h, in basal conditions (left panels) or in the presence of 100 µM H_2_O_2_ as a pro-oxidant stimulus (right panels). Data are the means ± SD of three different experiments. Values not sharing the same superscript letter are significantly different (*p* < 0.05, one-way ANOVA followed by Tukey’s test).

Plenty of evidence has contributed to demonstrate that ROS production plays a key role in the development of some inflammatory disorders, such as contact dermatitis induced by SDS [59]. For this reason, we analyzed ROS production in HaCaT (Figure 5) and NCTC 2544 (Figure 6) keratinocytes in the presence of SDS (Figure 5A and Figure 6A) or TNF-α (Figure 5B and Figure 6B). The treatment with DHA at both the concentrations used (10 and 30 µM) and in the free or encapsulated form did not modify ROS production in both the keratinocytes in basal conditions or in the presence of an exogenous pro-oxidant stimulus (H_2_O_2_, 100 µM) (Figure 5A,B and Figure 6A,B). This result was not surprising, since DHA, even if highly susceptible to lipid peroxidation, due to its chemical structure, was shown not to modify the oxidative cellular status in most normal cells [60]. On the contrary, it has been reported that DHA, especially if used at high concentrations, may induce a pro-oxidant cytotoxic effect in cancer cells, which are known to be defective in antioxidant defenses [60]. The treatment of keratinocytes with both SDS and TNF-α induced a significant increase in ROS production both in the absence (Figure 5A,B and Figure 6A,B, left panels) or in the presence (Figure 5A,B and Figure 6A,B, right panels) of H_2_O_2_. Particularly, in the HaCaT cells, free DHA was never able to significantly reduce ROS production, regardless of being treated with SDS or TNF-α. When DHA was enclosed in RV-SLNs, it showed a tendency to more efficaciously inhibit ROS production, but a significant effect was observed only with the concomitant treatments of both TNF-α and H_2_O_2_ (Figure 5B, right panels). In NCTC 2544 keratinocytes, similar results were obtained, but significance was reached in all the conditions, except when the cells were treated with SDS in the absence of H_2_O_2_ (Figure 6A, left panel).

The higher antioxidant efficacy of DHA-RV-SLNs could be in part explained by the presence of the powerful and well known antioxidant, RV, in the lipid matrix of the SLNs [61], which may protect DHA from its oxidative degradation and reduce the amplification of ROS production.

### 3.6. Effect of DHA and DHA-SLNs on the Activation of NLRP3 Inflammasome Induced by TNF-α in HaCaT Keratinocytes

It has previously been observed that NLRP3 inflammasome, a multiprotein complex which activates caspase-1 and induces the maturation and secretion of IL-1β, besides being activated by a series of danger intracellular signals (microorganisms and metabolism-derived products), may also be activated by an ROS intracellular increase [62]. Since, in our experimental model, we observed an increased IL-1β production (Supplementary Materials Figure S2), and a significant increase in ROS production (Figure 5 and Figure 6) following the TNF-α treatment, we analyzed whether such effects were related to NLRP3 inflammasome activation in the human keratinocytes. Moreover, we evaluated whether DHA, in its free form or carried by RV-SLNs, was able to modulate the expression of the different molecular components (the adaptor protein ASC, NLRP3, and cleaved caspase-1) of this multiprotein complex in these cells.

**Figure 7:**
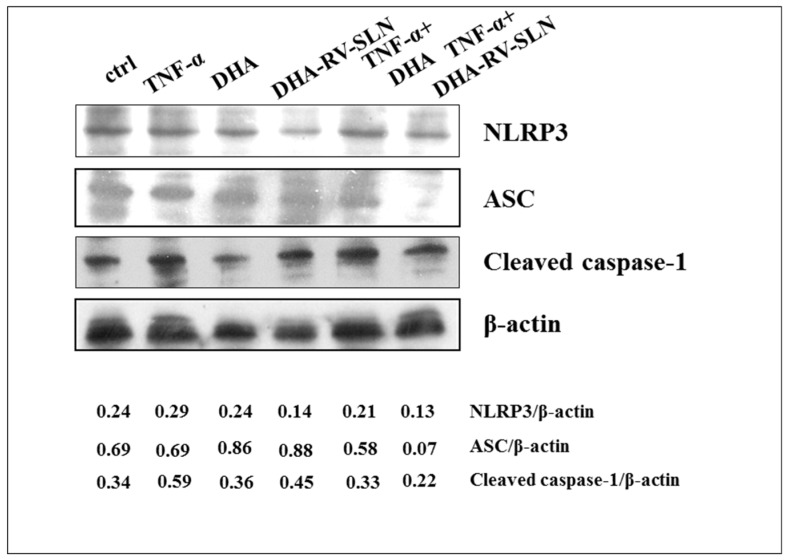
Effect of free DHA and DHA-RV-SLNs on the expression of NLRP3 inflammasome components. HaCaT cells were treated with free DHA (30 µM) and DHA-RV-SLNs (30 µM) in the absence or in the presence of TNF-α (50 ng/mL) for 24 h, and the expression of NLRP3, ASC, and cleaved caspase 1 was evaluated by Western blotting. A representative Western blot analysis of two different experiments is shown. The data represent the ratios between the densitometric values of the proteins and that of β-actin as a loading control.

Figure 7 reports the effect of a 24 h treatment with TNF-α (50 ng/mL), alone and in combination with DHA (30 µM) in its free form or carried by RV-SLNs on the expression of NLRP3, ASC, and cleaved caspase-1. We observed that TNF-α treatment was able to markedly increase the expression levels of cleaved caspase-1 and that free DHA was not able to modify the expression of any of the inflammasome proteins, either in the presence or absence of TNF-α. However, DHA encapsulated in RV-SLNs showed a remarkable inhibitory effect on the expression of NLRP3, ASC, and cleaved caspase-1, but only following theTNF-α stimulation. This result suggests that the encapsulation of DHA in RV-SLNs could enhance the anti-inflammatory properties of this fatty acid by an additional mechanism, i.e., by making it able to inhibit the NLRP3 inflammasome activation during the inflammatory process of the skin.

### 3.7. Effect of DHA and DHA-RV-SLNs on the Basal and TNF-α-Induced IL-1β Production in Cultured Keratinocytes

We also evaluated the effect of TNF-α on the production of IL-1β, IL-6, and MCP-1. TNF-α (50 ng/mL) induced a significant increase in IL-1β production both in HaCaT (Supplementary Figure S2A) and NCTC 2544 (Supplementary Materials Figure S2B) keratinocytes (HaCaT cells: 185.6% increase; NCTC 2544 cells: 173.1% increase vs. control, after 72 h of treatment). After 48 h of treatment, free DHA did not significantly inhibit the basal HaCaT cell cytokine production at any of the concentrations used. However, after 72 h, it was able to significantly reduce IL-1β production, but only at the highest concentration used (30 μM, 21.3% reduction vs. control, *p* < 0.05). DHA was able to significantly reduce the cytokine production more efficiently when enclosed in RV-SLNs (after 72 h of treatment: 30 µM DHA vs. TNF-α, 31.3% inhibition; 30 µM DHA-SLN vs. TNF-α, 77.1% inhibition, *p* < 0.05). Similar results were obtained in the NCTC 2544 keratinocytes. In this case, the inhibition exerted by free 30 µM DHA was 43.9% (*p* < 0.05) and 68.1% (*p* < 0.05) of that exerted by the same concentration of DHA-RV-SLNs.

### 3.8. Effect of DHA and DHA-RV-SLNs on the Basal and TNF-α-Induced IL-6 Production in Cultured Keratinocytes

We observed that free DHA was able to inhibit the production of IL-6 both in basal conditions and in the presence of TNF-α. This effect was observed both in HaCaT (Supplementary Materials Figure S3A) and in NCTC 2544 (Supplementary Materials Figure S3B) cells. As observed for IL-1β, DHA-RV-SLNs were significantly more powerful in inhibiting IL-6 production than free DHA, particularly in the presence of TNF-α (HaCaT cells, 72 h of treatment: 30 µM DHA vs. TNF-α, 52.4% inhibition; 30 µM DHA-RV-SLNs vs. TNF-α, 72.2% inhibition; NCTC 2544 cells, 30 µM DHA vs. TNF-α: 60.3% inhibition; 30 µM DHA-RV-SLNs vs. TNF-α: 87.7% inhibition, *p* < 0.05).

### 3.9. Effect of DHA and DHA-RV-SLNs on the Basal and TNF-α-Induced MCP-1 Production in Cultured Keratinocytes

The levels of MCP-1 (Supplementary Materials Figure S4) were significantly increased in the presence of TNF-α (after 72 h of treatment, HaCaT cells: About 45 fold increase, NCTC 2544: About 10 fold increase). In both HaCaT (Supplementary Materials Figure S4A) and NCTC 2544 cells (Supplementary Materials Figure S4B), DHA was always more efficient in inhibiting MCP-1 production when enclosed in RV-SLNs as compared to its free form (HaCaT cells, 72 h of treatment: 30 µM DHA vs. TNF-α, 48.6% inhibition; 30 µM DHA-SLN vs. TNF-α, 81.8% inhibition; NCTC 2544 cells, 30 µM DHA vs. TNF-α, 64.8% inhibition; 30 µM DHA-SLN vs. TNF-α, 93.8% inhibition, *p* < 0.05).

These results confirmed the anti-inflammatory activity of DHA, and further demonstrate that its beneficial effect may be enhanced through its encapsulation in SLNs with a lipid matrix containing RV-stearate. In particular, the inclusion of DHA in RV-SLNs markedly potentiated the anti-inflammatory effects of this fatty acid even at very low concentrations and could allow the use of very low amounts of DHA to obtain powerful anti-inflammatory effects. This possibility is noteworthy, particularly in view of the unsustainability of an intensive utilization of fish, the main natural source for the purification of these fatty acids, currently and largely used for human supplementation [63].

## 4. Conclusions

Overall, the data obtained demonstrated that the inclusion of the LC-ω-3 PUFA DHA into RV-SLNs significantly increased its ability to reduce the SDS-induced cytotoxic effect and to inhibit cytokine and ROS production induced by both SDS and TNF-α in keratinocytes. Moreover, they showed that DHA-RV-SLNs were able to modulate the expression of the NLRP3 inflammasome components, whose activation is responsible for IL-1β maturation and secretion. Since the encapsulation of DHA into RV-SLNs may represent a possibility to increase the levels of this fatty acid in cells, we suggest that a DHA-RV-SLN dietary supplementation could be useful for the prevention and cure of inflammatory skin disorders. In fact, it could ensure the sustained tissue availability of DHA independently from its frequent topical application and could result in a long-lasting protection of the skin from the pro-irritant and pro-inflammatory action of environmental hazards.

## Supplementary Materials

The following are available online at https://www.mdpi.com/2072-6643/11/6/1400/s1, Figure S1: Effect of SDS on cell cytotoxicity in HaCaT and NCTC 2544 keratinocytes, Figure S2: IL-1β production in HaCaT and NCTC 2544 keratinocytes treated with TNF-α alone and in combination with DHA delivered or not through RV-SLNs, Figure S3: IL-6 production in HaCaT and NCTC 2544 keratinocytes treated with TNF-α alone and in combination with DHA delivered or not through RV-SLNs, Figure S4: MCP-1 production in HaCaT and NCTC 2544 keratinocytes treated with TNF-α alone and in combination with DHA delivered or not through RV-SLNs.
