# Supplementary material for: Anti-Irritant and Anti-Inflammatory Effects of DHA Encapsulated in Resveratrol-Based Solid Lipid Nanoparticles in Human Keratinocytes
Source: Nutrients. 2019 Jun 21;11(6):1400. doi: 10.3390/nu11061400 (PMC6627705; doi:10.3390/nu11061400)
Supplement: Supplementary file 1 [file nutrients-11-01400-s001.pdf]

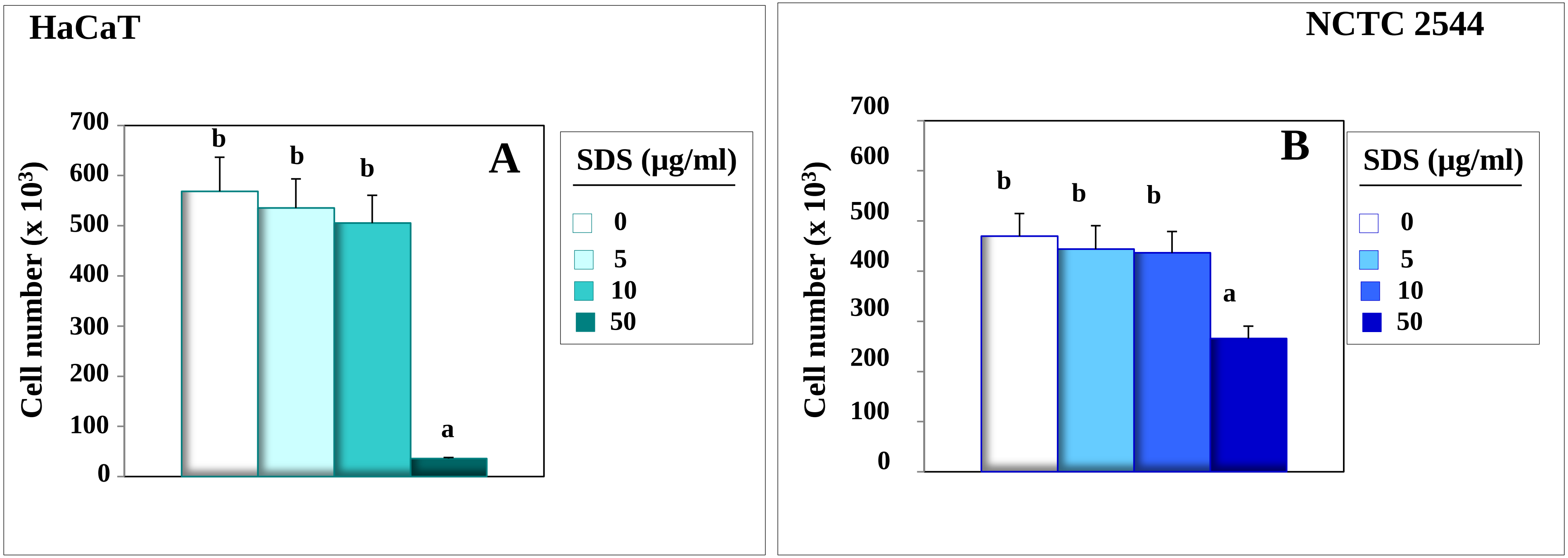

Supplementary Figure 1

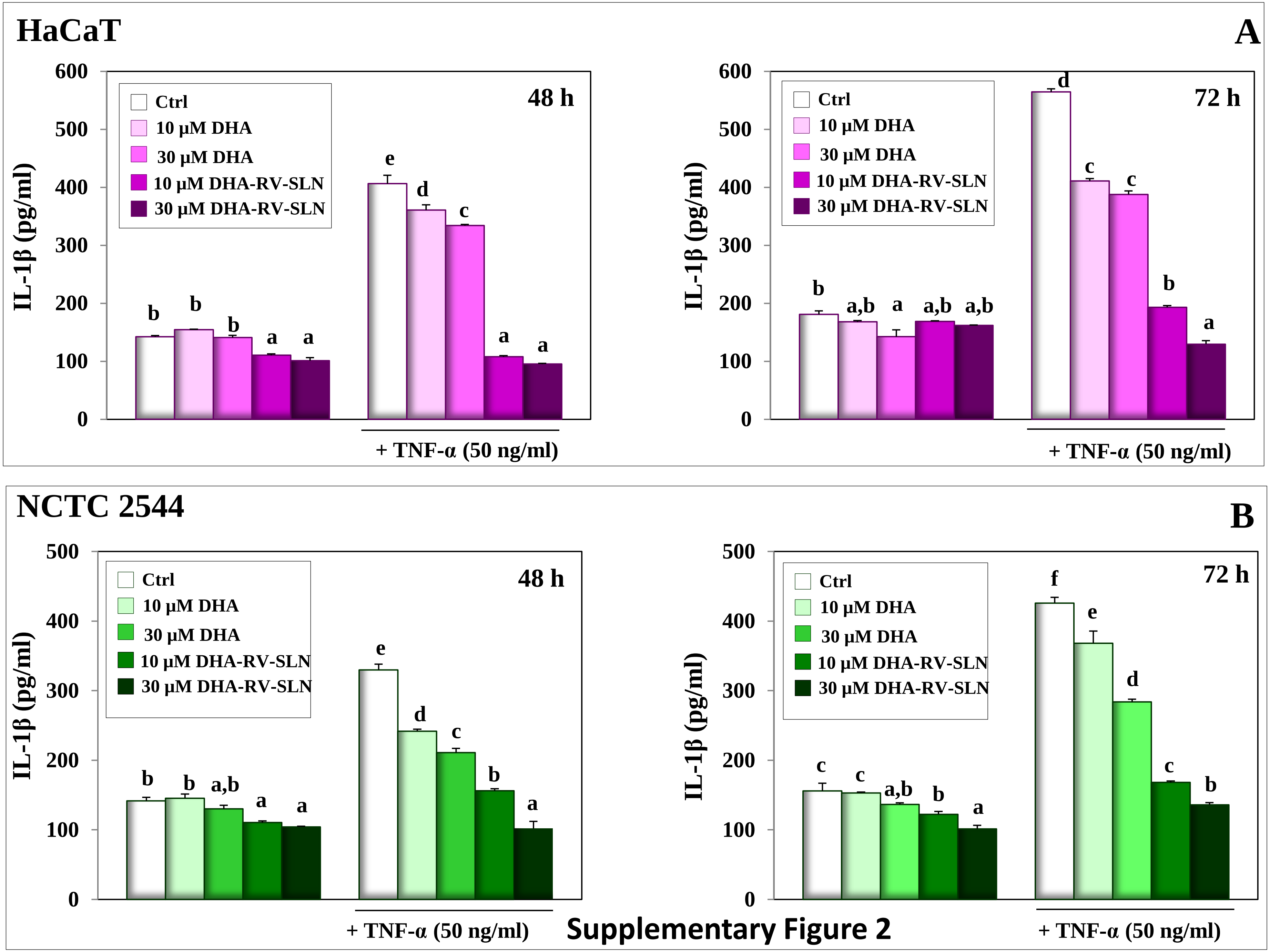

Supplementary Figure 2

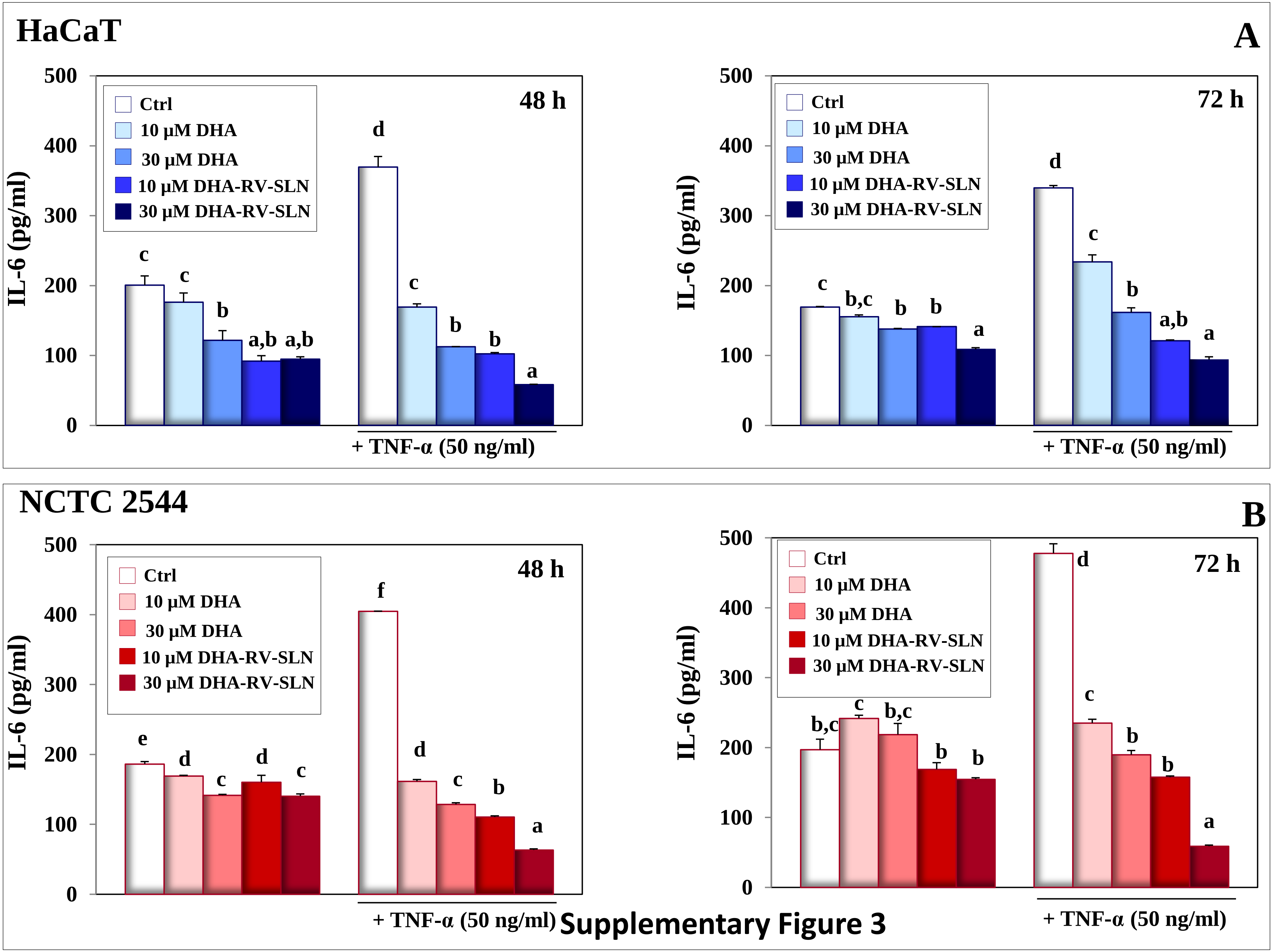

Supplementary Figure 3

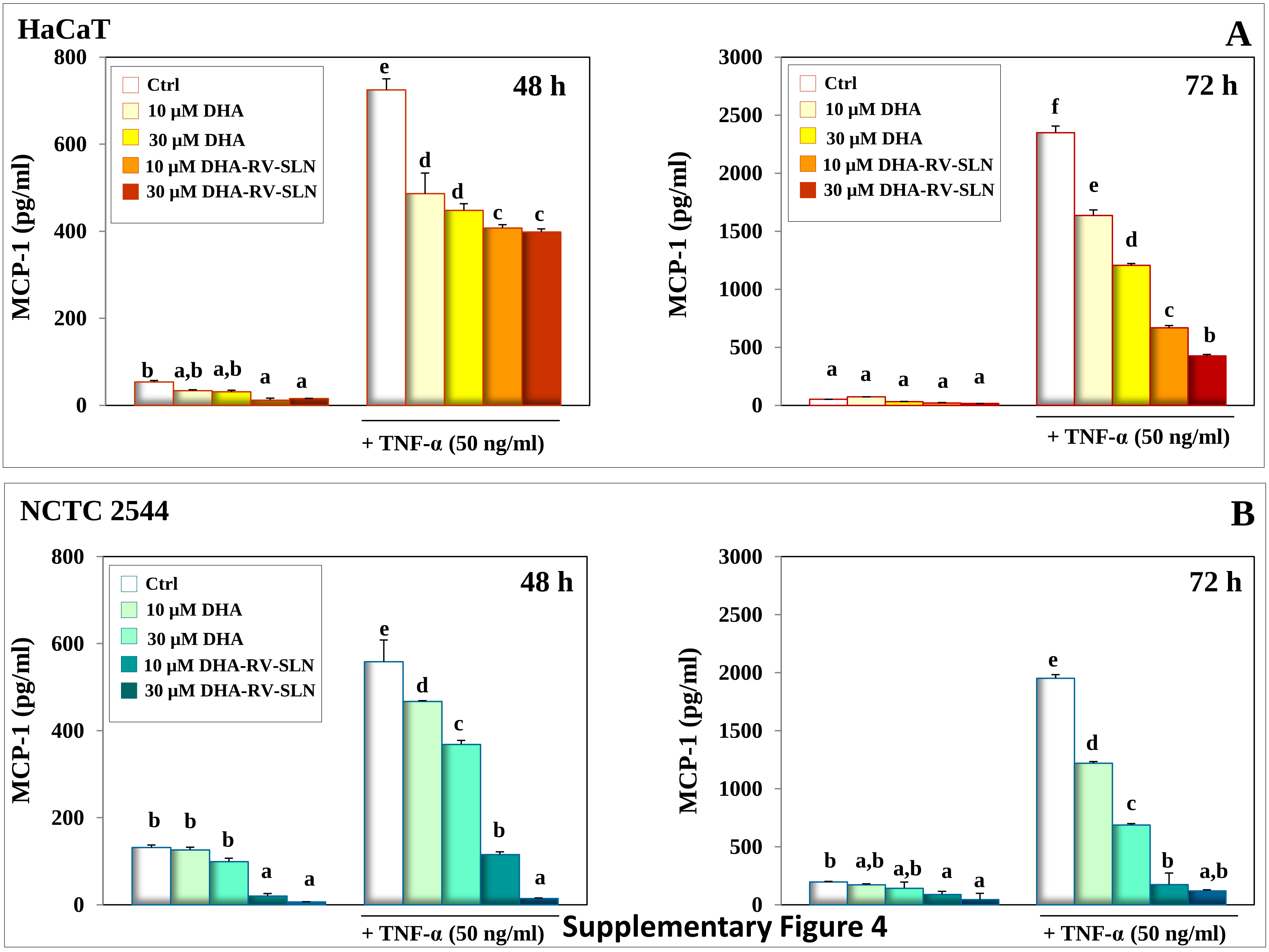

Supplementary Figure 4
